# Transcriptional Modulation by Idelalisib Synergizes with Bendamustine in Chronic Lymphocytic Leukemia

**DOI:** 10.3390/cancers11101519

**Published:** 2019-10-09

**Authors:** Sara E. F. Kost, Ali Saleh, Edgard M. Mejia, Marina Mostafizar, Eric D. J. Bouchard, Versha Banerji, Aaron J. Marshall, Spencer B. Gibson, James B. Johnston, Sachin Katyal

**Affiliations:** 1 Research Institute in Oncology and Hematology, CancerCare Manitoba, Winnipeg, MB R3E 0V9, Canada; skost@cancercare.mb.ca (S.E.F.K.); asaleh2@cancercare.mb.ca (A.S.); vbanerji1@cancercare.mb.ca (V.B.); spencer.gibson@umanitoba.ca (S.B.G.); 2 Department of Immunology, University of Manitoba, Winnipeg, MB R3E 0V9, Canada; mejiae@myumanitoba.ca (E.M.M.); Aaron.Marshall@umanitoba.ca (A.J.M.); 3 Department of Pharmacology and Therapeutics, University of Manitoba, Winnipeg, MB R3E 0V9, Canada; marinamostafizar@gmail.com; 4 Department of Environmental Health and Safety, Simon Fraser University, Burnaby, BC V5A 1S6, Canada; eric_bouchard@sfu.ca; 5 Department of Internal Medicine, University of Manitoba, Winnipeg, MB R3E 0V9, Canada; 6 Department of Biochemistry and Medical Genetics, University of Manitoba, Winnipeg, MB R3E 0V9, Canada

**Keywords:** chronic lymphocytic leukemia, bendamustine, idelalisib, drug combination therapy, DNA damage, transcriptional regulation

## Abstract

The phosphatidyl-inositol 3 kinase (PI3K) δ inhibitor, idelalisib (IDE), is a potent inhibitor of the B-cell receptor pathway and a novel and highly effective agent for the treatment of chronic lymphocytic leukemia (CLL). We evaluated the activities of IDE in comparison to bendamusine (BEN), a commonly used alkylating agent, in primary CLL cells ex vivo. In contrast to BEN, IDE was cytotoxic to cells from extensively-treated patients, including those with a deletion (del)17p. Cross-resistance was not observed between BEN and IDE, confirming their different modes of cytotoxicity. Marked synergy was seen between BEN and IDE, even in cases that were resistant to BEN or IDE individually, and those with deletion (del) 17p. CD40L/interleukin 4 (IL4) co-treatment mimicking the CLL microenvironment increased resistance to IDE, but synergy was retained. PI3Kδ-deficient murine splenic B cells were more resistant to IDE and showed reduced synergy with BEN, thus confirming the importance of functional PI3Kδ protein. Although IDE was observed to induce γH2AX, IDE did not enhance activation of the DNA damage response nor DNA repair activity. Interestingly, IDE decreased global RNA synthesis and was antagonistic with 5,6-Dichlorobenzimidazole 1-b-D-ribofuranoside (DRB), an inhibitor of transcription. These findings add to the increasingly complex cellular effects of IDE, and B cell receptor (BCR) inhibitors in general, in CLL.

## 1. Introduction

Most patients with chronic lymphocytic leukemia (CLL) will require therapy at some point during their disease course, and typically receive chemoimmunotherapy as initial treatment [1]. Standard treatments include alkylating agents (chlorambucil, CLB; bendamustine, BEN), or nucleoside analogues (fludarabine, FLU), alone or in combination with an anti-CD20 monoclonal antibody [1]. BEN is a bifunctional alkylating agent with a mechanism unique from other alkylating agents, such as CLB [2,3]. We and others have previously demonstrated that BEN is synergistic with FLU in primary CLL cells, and this synergy was related to increased DNA damage [2,4]. 

However, the algorithm for therapy has recently shifted, with the advent of novel targeted therapies [1,5]. These therapies include the B cell receptor (BCR) pathway inhibitors, ibrutinib (IBR) and idelalisib (IDE), which target Bruton’s tyrosine kinase (BTK) and the δ isoform of phosphatidyl-inositol 3 kinase (PI3Kδ), respectively [1]. These agents have a unique mechanism of action, and are presently used in patients who have relapsed following chemoimmunotherapy or have a deletion (del) 17p, which is typically associated with a p53 mutation [1]. PI3Kδ is one of four isomers of the Class I PI3Ks, and is composed of a catalytic subunit p110 and regulatory subunit p85. Following activation of the BCR, PI3K becomes phosphorylated by Syk and phosphorylates phosphatidylinositol-4,5-bisphosphate (PIP2) to phosphatidylinositol-3,4,5-trisphosphate (PIP3), leading to activation of protein kinase B (AKT) [6]. PI3Kδ activity is higher in CLL cells than in normal cells, and IDE induces apoptosis in CLL cells in vitro by inhibiting the BCR pathway [6,7,8,9]. In addition, these inhibitors reduce CLL cell adherence to stromal cells in the microenvironment [7,8]. When combined with rituximab, either in the first or second line settings [10,11,12,13,14], IDE has significant activity in CLL, and autoimmune toxicity was seen more commonly in the front line setting (hepatitis, colitis and pneumonitis) [15]. IDE has also been combined with chemotherapy, to improve antitumor activity and specificity. A placebo-controlled Phase III clinical trial of IDE with BEN/rituximab (BR) showed higher clinical efficacy than with BR alone, but with significantly more infections and marrow suppression, demonstrating a lack of tumor specificity [16]. In vitro studies have also suggested synergy between IDE and BEN, although the mechanism remains unclear [17,18]. 

In the present study we have evaluated the cytotoxicity of IDE in primary CLL cells ex vivo, demonstrating a lack of cross-resistance between this agent and chemotherapy, significant cross-resistance between IDE and IBR and a decrease in IDE activity when the microenvironment was simulated with CD40L/IL4. There was synergy between IDE and FLU, CLB or BEN, particularly in the presence of CD40L/IL4. Interestingly, synergy between BEN and IDE appeared unrelated to enhanced DNA damage but rather via transcriptional modulation. Our findings suggest a novel biological role for IDE in anti-CLL therapy. 

## 2. Results

### 2.1. IDE Is Cross-Resistant with IBR but Not Chemotherapeutic Agents and Displays Synergy with Chemotherapeutics, Incubated Alone or with CD40/IL4 

**Figure 1:**
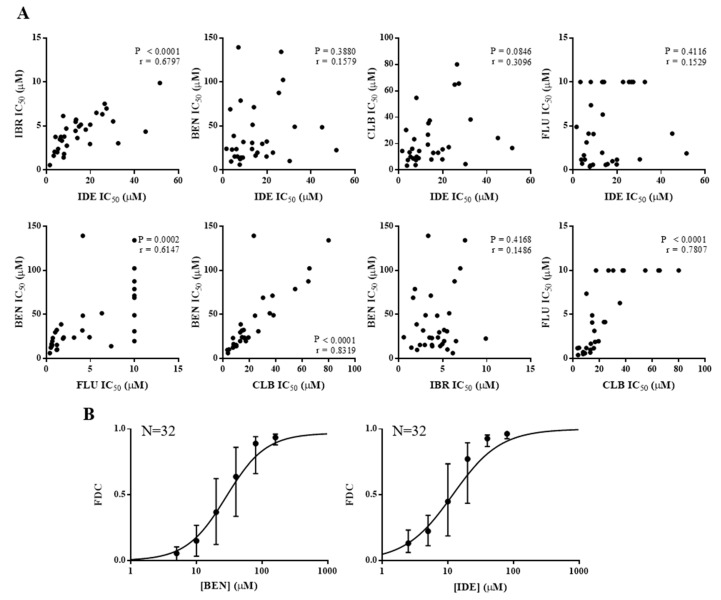
Idelalisib (IDE) is not cross-resistant with chemotherapies bendamustine (BEN), chlorambucil (CLB), or fludarabine (FLU) but is cross-resistant with ibrutinib (IBR), and IDE and BEN have similar sigmoidal dose-response curves. (**A**) Correlation of the concentration required to inhibit cell viability by 50% (IC_50_) between the different chronic lymphocytic leukemia (CLL) drugs. IC_50_s were calculated 72 h post drug treatment of primary CLL samples. (**B**) Dose-response curves of the median and interquartile range of 32 unique primary CLL samples treated with single agent BEN or IDE for 72 h (FDC = fraction of dead cells).

To determine the relative sensitivities of CLL cells to IDE, as compared to IBR and chemotherapy, 32 unique CLL patient cell isolates were treated with drug for 72 h and cytotoxicity measured (Figure 1A, Table 1). The dose-response curves of CLL samples treated with IDE or BEN demonstrated a sigmoidal curve with both agents (Figure 1B). IBR also showed a similar sigmoidal curve (not shown). The dose required to inhibit cell viability by 50% (IC_50_) varied significantly (32-fold) for IDE (1.6–51.6 μM, median 13.3 μM), while the variation was less (16-fold) for IBR (0.6–9.9 μM, median 4.1 μM). Cells from four patients with del 17p were sensitive to treatment with these agents (Table 1, as determined by comparison of IC_50_ values from samples with or without a del 17p). There was a significant correlation between the IC_50_ values of IDE and IBR (Figure 1A, *p* < 0.0001), demonstrating cross-resistance and similar mechanisms of action, consistent with their function as inhibitors of the BCR pathway. In contrast, there was no correlation between the IC_50_ values of IDE and BEN (*p* = 0.39), IDE and CLB (*p* = 0.085), or IDE and FLU (*p* = 0.41; Table 1, Figure 1A). However, as we have shown previously [2], significant cross-resistance was observed between the chemotherapeutic agents, with the IC_50_ values of the drugs significantly correlating with each other (BEN:CLB *p* < 0.0001, BEN:FLU *p* = 0.0002, CLB:FLU *p* < 0.0001). Moreover, in contrast to IBR and IDE, cells from the patients with a del 17p were resistant to BEN and the other chemotherapies (Table 1).

**Figure 2:**
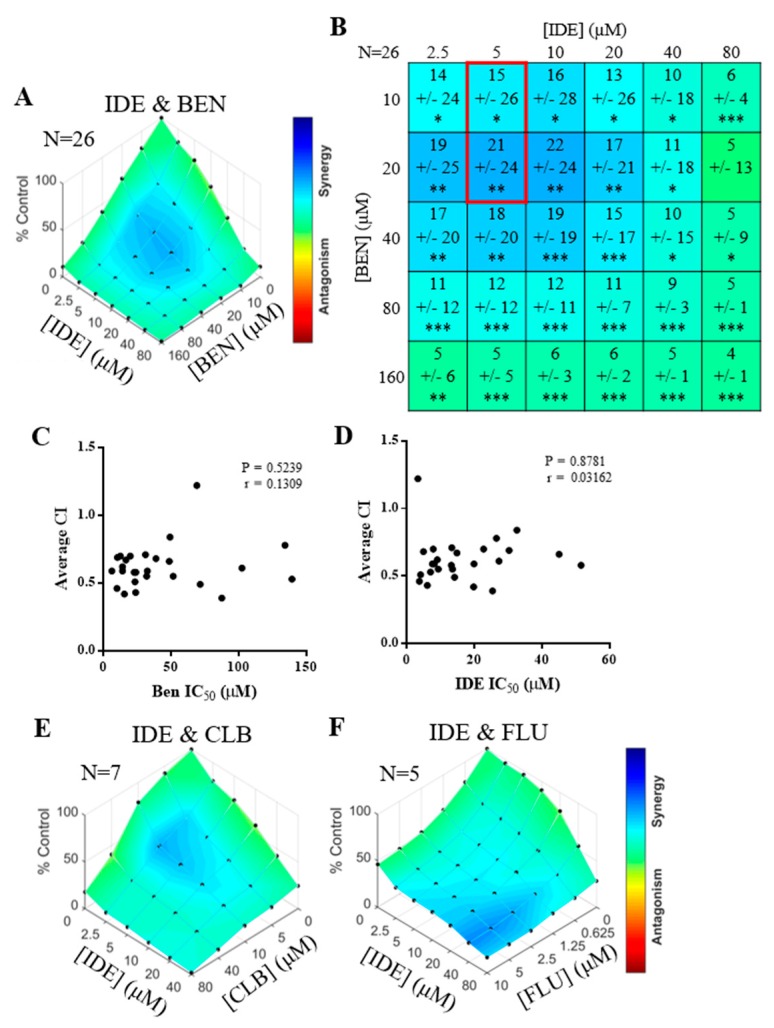
Idelalisib (IDE) is synergistic with bendamustibe (BEN) and other chemotherapies. Cell death in primary chronic lymphocytic leukemia (CLL) samples 72 h post drug treatment. (**A**) Combenefit synergy plots from combining BEN and IDE representing the average difference in cell death compared to that predicted by the single dose-response curves for each agent in 26 CLL samples. Blue—synergy, green—additivity and, red—antagonism (**B**) Statistical table from A with 95% confidence intervals (* *p* < 5 ×10^−2^_;_ ** *p* < 10^−3^, *** *p* < 10^−4^) highlighting the clinically relevant concentrations (red box). (**C**,**D**) Graphs showing the correlation between the drug concentration required to inhibit cell viability by 50% (IC_50_) of BEN (**C**) or IDE (**D**) to the average combination index (CI) value of the clinically relevant single agent concentrations (red box in **B**). (**E***,***F**) Combenefit synergy plots showing synergy between IDE and chlorambucil (CLB) (**E**) or fludarabine (FLU) (**F**).

To determine if synergy was observed between the BCR pathway inhibitors and chemotherapy, as previously suggested [17], a matrix of dose combinations was created and observed cell death was compared to predicted cell death [19]. In 26 unique primary CLL samples, significant synergy was observed when combining BEN with IDE, using clinically relevant doses of each agent (5 µM for IDE and 10–20 µM for BEN; Figure 2A,B, red box) [20]. While the degree of synergy (as measured by the average combination index (CI) value at the clinically-relevant doses; see Section 4.12 of Materials and Methods) varied between patients, it was observed equally in patients who were resistant or sensitive to BEN or IDE (*p* = 0.5239 and *p* = 0.8781 for IDE and BEN, respectively; Figure 2C,D, Table 1). Thus, combining these agents may overcome single-agent resistance. The degree of synergy between BEN and IDE was not enhanced by prolonging drug exposure and was similar with 24, 48, and 72 h drug treatments (data not shown). 

To test if the synergy between IDE/BEN was unique to BEN, IDE was combined with CLB or FLU. Similarly, IDE displayed synergy with CLB and FLU (Figure 2E,F). At clinically-relevant concentrations for each drug [20] the degree of synergy for the IDE/CLB combination was equivalent to the BEN/IDE combination (median combination index (CI) for CLB/IDE and BEN/IDE = 0.59, and 0.56, respectively), but greater synergy was seen between IDE/FLU (median CI for FLU/IDE = 0.27). The pattern and degree of synergy observed was consistent in samples from three patients, treated with the combinations of IDE/BEN, IDE/CLB, and IDE/FLU (data not shown). 

**Figure 3:**
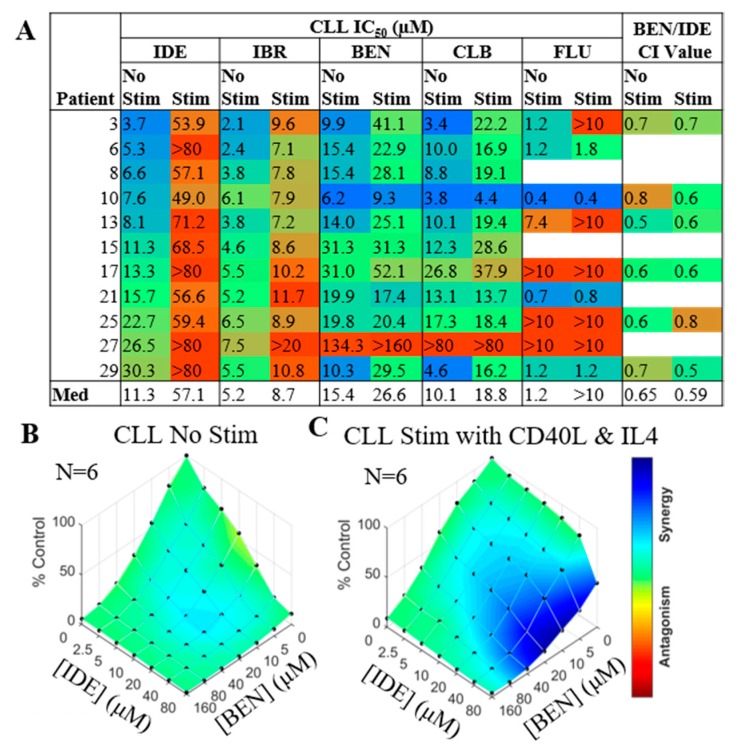
Enhanced synergy occurs between idelalisib (IDE) and bendamustine (BEN) when the microenvironment is simulated by the addition of CD40L and interleukin 4 (IL4). Cell death was measured 72 h post drug treatment. (**A**) Table showing differences in response of primary chronic lymphocytic leukemia (CLL) samples to single agent drugs (blue—sensitivity, red—resistance) or BEN/IDE combination index (CI) value at clinically relevant concentrations (blue—synergy, green—additivity and, red—antagonism) with or without stimulation. (**B**,**C**) Combenefit synergy plots combining BEN and IDE in 6 primary CLL patient cells without (**A**) or with (**B**) stimulation with CD40L/IL4. Blue—synergy, green—additivity and, red—antagonism.

CD40/ interleukin 4 (IL4) co-treatment of CLL patient cells has previously been shown to be a highly-effective and consistent method to simulate the CLL microenvironment [21,22]. CLL cells derived from 11 patients were pre-treated for 1 h with either serum-free hybridoma media (SFM) alone or with CD40/IL4. The IDE IC_50_ values increased in all patients following the addition of CD40L/IL4; four patients became IDE resistant (Figure 3A). All samples increased in their IBR LD_50_ values, but not to the extent as with IDE treatment. In contrast, the BEN, CLB and FLU IC_50_ values did increase in some patients but remained similar for others (Figure 3A). Furthermore, BEN/IDE synergy was compared in six unique primary CLL samples against CD40L/IL4 stimulation. While a high degree of synergy was only observed with stimulation using high doses of IDE (Figure 3 B,C), a lower degree of synergy was also observed using clinically-relevant concentrations of these two agents [20] (median CI value of 0.59 or 0.64 with or without stimulation, respectively; Figure 3A). 

### 2.2. IDE and BEN Are Also Synergistic in Normal B Cells but Not T Cells 

**Figure 4:**
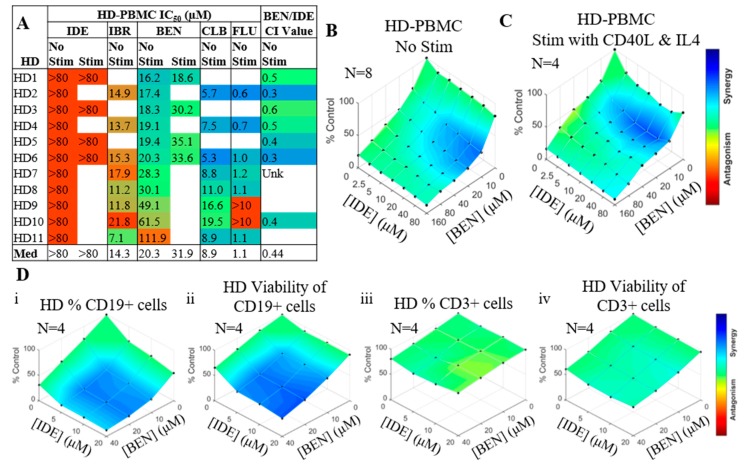
Idelalisib (IDE)/ bendamustine (BEN) are synergistic in non-chronic lymphocytic leukemia (CLL) donor peripheral blood mononuclear cells (PBMCs). (**A**) Table showing differences in response of age matched non-CLL healthy donor (HD)-PBMCs to single agent drugs or BEN/IDE combination index (CI) value. Unk = unknown. (**B**,**C**) IDE and BEN synergy in HD-PBMCs without (**B**) or with (**C**) CD40L/interleukin 4 (IL4) stimulation. (**D**) BEN/IDE synergy in i) the number of, or ii) the viability of the HD-CD19+ cells, and iii) the number of, or iv) the viability of the HD-CD3+ cells.

To determine if the synergy between IDE and BEN was CLL-specific, peripheral blood mononuclear cells (PBMCs) were isolated from age-matched healthy donors (HD) and BEN/IDE synergy was tested with and without CD40L/IL4 stimulation. As expected, since the composition of PBMCs in HD is mainly T cells, all PBMC samples were resistant to single agent IDE (Figure 4A), while responses to BEN, CLB, and FLU were similar to the CLL samples. PBMCs required more IBR than the CLL samples to reach the IC_50_ (Figure 4A). Synergy was observed between BEN and IDE in the PBMCs with the greatest synergy observed at the highest (IDE) (Figure 4B). At clinically-relevant concentrations, the median CI value was 0.44, close to that of the CLL samples at 0.59 (Table 1, Figure 4A). Upon stimulation with CD40L/IL4, PBMCs resistance to IDE was maintained (IC_50_ > 80 µM), while the response to BEN was decreased slightly (from IC_50_ values of 20.3 µM to 31.9 µM) and the synergy shifted to lower (IDE) (Figure 4A–C). To determine which cells within the PBMCs were responding to the treatment, co-staining was performed with annexin V/7-aminoactinomycin D (AV/7AAD), CD19 and CD3. Synergy was observed in both the number of and viability within the CD19+ but not CD3+ populations, indicating that BEN and IDE exhibit B cell-specific synergistic cytotoxicity (Figure 4D).

### 2.3. B Cells from Mice Lacking Functional PI3Kδ Display Reduced IDE Sensitivity and Synergy with BEN/IDE

To determine the influence of PI3Kδ on IDE sensitivity and IDE/BEN synergy, splenic B cells isolated from mice lacking functional PI3Kδ protein were exposed to single agent IDE, BEN, CLB, FLU, or IBR for 72 h. B cells from the PI3Kδ-deficient mice displayed significantly reduced response to IDE at 72 h (IC_50_, 20.1 µM) compared to wild-type (WT) counterparts (IC_50_ of 1.7 µM), however, the residual response suggests additional PI3Kδ-independent molecular targets of IDE (Figure S1A,C). As expected, the responses to the other agents changed minimally between cells from the two mouse types (Figure S1B,C). At clinically-relevant concentrations for humans [20], B cells from the PI3Kδ-deficient mice displayed additivity with BEN/IDE (average CI values of 0.93 and 0.95 at 18 and 72 h, respectively) whereas WT B cells displayed synergy with BEN/IDE, increasing from 18 to 72 h (average CI values of 0.73 and 0.60 at 18 and 72 h, respectively; Figure S1C). Thus, PI3Kδ is required for IDE activity and for synergy between IDE and BEN.

### 2.4. IDE Alone Induces γH2AX Formation but Not DNA Breaks, and Does Not Inhibit DNA Damage Repair in CLL Cells

It has been reported that IDE can induce γH2AX formation, and we have evaluated whether this is related to the cytotoxicity of this agent [17]. We integrated γH2AX analysis, an indicator for DNA double-stranded breaks, with AV/7AAD after treating cells with the drugs for 18 h. IDE produced a small but significant increase in γH2AX, which increased with higher drug concentrations (Figure S2A,B), but no breaks by comet assay (data not shown). However, despite the induction of cell death, IDE produced less γH2AX than IR or BEN as a single agent, regardless of dose, indicating that DNA damage was not a primary cytotoxic mechanism for IDE. γH2AX analysis following CLL cell treatment with IDE combined with IR revealed that the synergy between IDE and chemotherapy was not paralleled by an increased production of γH2AX (Figure S2C,D). Similarly, analysis at 18 h post IDE and BEN treatment for viability, γH2AX and double strand breaks (DSBs) indicated that γH2AX levels were minimal and no DNA breaks (comet tails) were observed with IDE or BEN alone or with the combination (Figure S2E–G). 

To determine if IDE could inhibit DNA repair, CLL cells were treated with 10 Gy irradiation and DNA breaks were sequentially assessed by γH2AX levels and comet assay, in the presence and absence of 10 μM IDE (Figure S2H,I) [23]. DNA damage analysis of CLL cells treated with 10 μM IDE for 18 h, either alone or combined with CD40L/IL4, showed no IDE-dependent change in the number of DNA breaks Compared to cells incubated in media alone, cells incubated with CD40L/IL4 appeared to have less breaks, regardless of the presence of IDE. In contrast to γH2AX disappearance (Figure S2H), repair of the DSB began within 30 min and 50% of breaks were repaired by 18 h (Figure S2I). Similarly, DNA repair appeared to be more complete at 18 h in cells incubated with CD40L/IL4 independently of IDE. While levels of the DNA damage response (DDR) protein, Ataxia-Telangiectasia mutated (ATM), were slightly increased with stimulation with CD40L/IL4 [24], IDE did not appear to alter baseline levels of ATM or transcriptional intermediary factor 1β (TIF1β) nor affect activation of the ATM dependent DDR response (Figure S3). Combined, these data further support the concept that the observed synergistic cytotoxicity between IDE and BEN was unrelated to increased DNA damage or repair deficiency.

### 2.5. IDE Induces γH2AX Formation Independent of PI3Kδ

**Figure 5:**
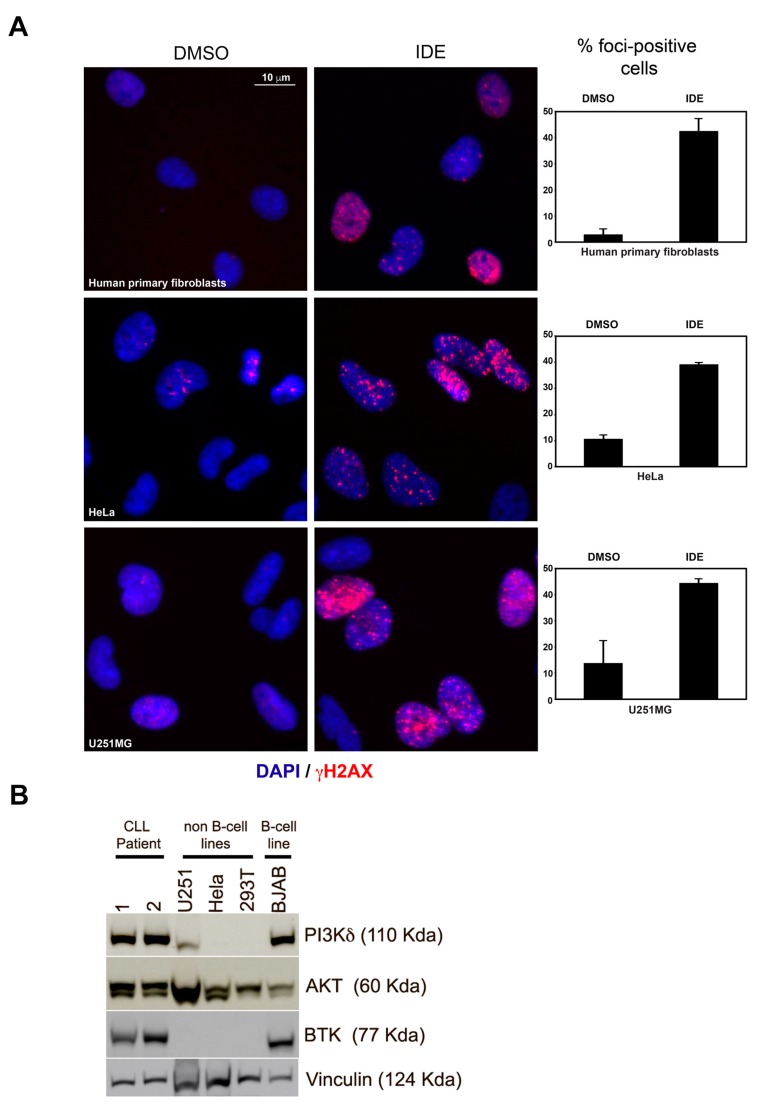
Induction of γH2AX foci by idelalisib (IDE) in non-B cell lineage cell lines. (**A**) Human primary fibroblasts, HeLa and U251MG cells were treated with IDE (20 µM, in media) for 18 h followed by immunostaining for γH2AX and imaged via epifluorescence microscopy. In the absence of an intact B cell receptor (BCR) signaling pathway, IDE can induce phosphorylation of H2AX on ser-139. Graphs represent quantification of percentage γH2AX foci-positive cells (*n* = 100 for each treatment group, N = 3, error bars represent standard error of the mean (SEM), scale bar represents 10 μm and corresponds to all images). (**B**) Western blot showing absence of the δ isoform of phosphatidyl-inositol 3 kinase (PI3Kδ)—Bruton’s tyrosine kinase (BTK) signaling in non B-cell cell lines, compared to chronic lymphocytic leukemia (CLL) patients and B-cell cell lines.

To determine whether BCR activation is required for the IDE-dependent effects on γH2AX, we interrogated adherent non-B cell lineage cells (human primary fibroblasts, HeLa, and U251MG), which lack BCR signaling (Figure 5A). In each of these PI3Kδ/BTK-negative cell lines, we observed significant γH2AX formation (Figure 5B) but no DNA damage, as measured by comet assay (data not shown). The apparent incongruence between γH2AX formation and DNA breaks indicates a DNA damage-independent cause for γH2AX formation, such as histone/chromatin alterations [25]. Furthermore, these data combined with the above CLL and murine studies support the assertion of additional unknown IDE-responsive, but PI3Kδ-independent molecular targets/activity. 

### 2.6. Synergy between BEN and IDE is Related to Changes in Transcription by IDE

**Figure 6:**
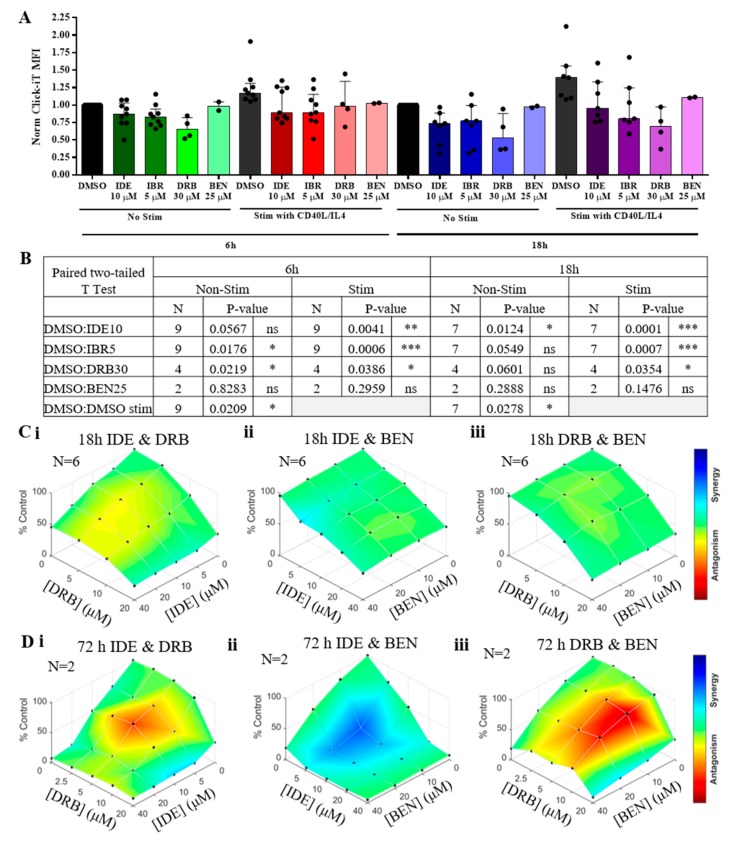
CD40/ interleukin 4 (IL4) increases DNA transcription in chronic lymphocytic leukemia (CLL), which is inhibited by idelalisib (IDE) and ibrutinib (IBR) but not by bendabustine (BEN). (**A**) Changes in global transcriptional activity in primary CLL cells 6 or 18 h post drug treatment. Click-iT® was measured in viable cells and samples were normalized (Norm) to the dimethyl sulfoxide (DMSO) control for each time-point (black bars). Bars represent median and interquartile range and dots represent individual samples. (**B**) Statistical table from **A**. not significiant (ns) = *p* > 0.05, * = *p* ≤ 0.05, ** = *p* ≤ 0.01 and *** = *p* ≤ 0.001 (**C,D**) Average Combenefit plots of i. 5,6-Dichloro-1-β- d-ribofuranosylbenzimidazole (DRB) and IDE, ii. IDE and BEN, or iii DRB and BEN in 6 primary CLL patients at 18 h post drug treatment (**C**) or in 2 primary CLL patients 72 h post-drug treatment (**D**). Blue—synergy, green—additivity and, red—antagonism.

Alterations in chromatin architecture is well-documented to modulate transcription and gene expression [26]. Therefore, we measured RNA synthesis in IDE-treated CLL patient samples to determine whether IDE has a direct role in modulating gene transcription. Analysis of newly generated RNA demonstrates that transcription is increased in CLL cells with the addition of CD40L/IL4 while treatment with 5,6-Dichlorobenzimidazole 1-b-D-ribofuranoside (DRB), a potent RNA Pol II inhibitor (negative control), significantly reduced RNA synthesis (Figure 6A,B; >50% repression after 18 h DRB treatment). Interestingly, IDE also significantly inhibited transcription, particularly when these cells were co-stimulated with CD40L/IL4. Similar results were obtained with IBR, indicating a generalized role for BCR inhibitors in regulating global RNA synthesis and gene expression in CLL (Figure 6A,B). Conversely, BEN had little effect on global transcription.

To determine if the synergistic cytotoxicity of IDE/BEN was related to alterations in transcription by IDE, we examined combinations of IDE, BEN and DRB. When cytotoxicity was assessed after 18 h drug treatment, IDE was antagonistic with DRB, indicating that these agents likely affect common molecular targets, particularly with short-term treatment (Figure 6C). Similarly, BEN was antagonistic with DRB at 72 h, indicating that transcription was required for the induction of cell death by BEN and was consistent with the fact that there was very little cell death at 18 h with BEN alone. In contrast, marked synergy was seen between BEN and IDE at 72 h, indicating that IDE induces specific changes in RNA expression, rather than a global reduction in transcription. 

## 3. Discussion

We have demonstrated that inhibition of PI3Kδ by IDE is cytotoxic to primary CLL cells, with the sensitivity of cells differing to that of the standard chemotherapeutic agents, BEN, CLB and FLU. Indeed, some patients who were previously treated with chemoimmunotherapy showed sensitivity to IDE, consistent with clinical experience [12,13]. Moreover, as previously observed ex vivo and in clinical studies, cells with a del 17p (p53 mutation), were equally sensitive to IDE [7,10,14]. Interestingly, sensitivity to IDE correlated closely with sensitivity to IBR, suggesting a lack of cross-resistance in these patients. However, we did not specifically evaluate IDE in CLL cells from patients that had become resistant to IBR. CD40/IL4 caused significant resistance to IDE, consistent with the potent effect of CD40L on PI3Kδ expression, and had a lesser effect on sensitivity to IBR and chemotherapy. 

Previous studies have demonstrated synergy between IDE and BEN [17], IBR and BEN [18], or MK2206 (an inhibitor of AKT) and BEN [27]. Similarly, we have confirmed that IDE is synergistic with BEN, CLB and FLU in primary CLL cells occurring even in patients who were resistant to one agent. Interestingly, synergy was also observed in cells exposed to CD40/IL4, suggesting that the effect would be observed in the microenvironment, as well as in the peripheral blood. Synergy required the inhibition of PI3Kδ, as it did not occur in the lymphocytes of C57 BL/6 mice with dysfunctional enzyme. In addition, the synergy was not CLL specific, as it was also seen in normal peripheral blood B cells, but not in T cells. 

A number of mechanisms may account for this synergy. First, there might be an interaction between the BCR and DNA repair pathways, although we did not observe an effect of BEN on p-AKT levels or of IDE on p-ATM production. Second, synergy may be related to increased BEN-induced DNA damage by IDE. However, this also appeared unlikely, as the combination of BEN and IDE did not produce a synergistic increase in γH2AX formation, to parallel the changes seen in cytotoxicity. Third, the synergy may be related to alterations in transcription by IDE. We observed transcriptional suppression with IDE and IBR treatments in CLL cells in vitro, which was most marked when RNA synthesis was initially increased by CD40/IL4. Similarly, a recent study in mantle cell lymphoma also showed that IDE can inhibit protein synthesis which correlated with a reduction in cell size and growth [28]. While a detailed analysis of the genes effected by IDE in CLL has not yet been carried out, a recent clinical study has demonstrated that the CLL cells of patients receiving IBR show primarily a decrease in genes involved in receptor/cytokines signaling and those expressed in proliferating cells, although increased expression of a subset of genes was also observed [29]. Interestingly, in contrast to the synergy seen with IDE, the global transcription inhibitor DRB produced antagonism with BEN indicating that inhibition or induction of specific proteins was required for the synergy between BEN/IDE. In this regard, it has been demonstrated that IDE can reduce Mcl-1 levels in CLL cells stimulated with anti-IgM antibody in vitro, although the degree of protection is inconsistent [17,30]. CD40/IL4 may preferentially induce Bcl_xL_ and Bfl-1 expression over Mcl-1 in CLL, and these are also likely reduced by inhibition of the BCR pathway [30]. Finally, IDE may reduce cell membrane levels of the activation marker CD69 in CLL, and CD69 has been associated with drug resistance [18]. 

Our comprehensive analysis confirmed that IDE alone produces a measurable increase in γH2AX in CLL cells, suggesting the induction of DNA double strand breaks [23,25]. ATM is autophosphorylated upon DNA double strand break induction, and phosphorylates serine 139 on H2AX to form γH2AX; Mediator Of DNA Damage Checkpoint 1 (MDC1) then binds to γH2AX, to prolong its half-life whereby γH2AX plays a key role in subsequent DNA repair [25,31,32]. However, recent studies have demonstrated that γH2AX has multiple functions apart from DNA repair, including roles in cell division, development and senescence [31]. Surprisingly, despite the increase in γH2AX with IDE, we did not detect DNA breaks by comet assay, indicating either that there were insufficient breaks for detection or that IDE may directly induce γH2AX formation. IDE might induce small numbers of DNA breaks in CLL cells by inhibiting the repair spontaneously formed DNA breaks, as has been observed with 2’-deoxycoformycin (pentostatin) [33]. However, this appears unlikely, as IDE did not inhibit the repair of irradiation-induced DNA breaks, as measured by γH2AX removal or comet assay. DNA breaks could also be induced by IDE through effects of the drug on activation-induced cytidine deaminase (AID). IDE increases the transcription of activation-induced cytidine deaminase (AID) in mouse B cells grown in CD40L/IL4, leading to class switch recombination and somatic hypermutations at the immunoglobulin gene site, and off-target translocations across the genome [34]. However, γH2AX formation with IDE was also observed in a variety of non-lymphoid cell lines (fibroblasts, HeLa cells and U251MG cells) indicating that the effect does not require a functional BCR PI3Kδ signaling axis. A similar effect in CLL cells was seen with IBR, and patients receiving IBR have demonstrated histone modifications, with loss of H3K27ac and H3K27me3, and changes in transcription, reflecting both increased and decreased RNA synthesis for different genes [29,35]. We cannot exclude the possibility that chromatin modifications may utilize short-lived DNA strand break intermediates that require γH2AX to maintain DNA integrity. It is noteworthy that Singh et al (2015) recently demonstrated interplay of high mobility group AT-hook 2 (HMGA2), ATM, and H2AX in transcription initiation [36]. However, McManus et al. (2005) characterized the occurrence of ATM-dependent but damage-independent γH2AX phosphorylation in all phases of the mammalian cell cycle [37]. Whether γH2AX expression increases in CLL cells after clinical treatment and whether histone modification occur following in vitro treatment requires further study [31]. As our IDE/DRB data indicates the involvement of specific transcriptional targets, future work would involve determining whether IDE alters H3K27ac and H3K27me3 levels at specific gene loci. ChipSeq studies comparing transcriptional events accompanying IDE-mediated transcriptional activation and/or repression would pinpoint new IDE-responsive targets. Furthermore, as we identified IDE-dependent but PI3Kδ-independent changes in γH2AX, which we inferred as IDE-induced chromatin modulation events, ChipSeq analysis using splenic cells from PI3Kδ-deficient/proficient mice would glean IDE drug on-target versus off-target effects; data with profound significance to CLL patients experiencing BCR drug toxicity and/or resistance.

In summary, we have demonstrated marked synergy between IDE and BEN in CLL cells, both when quiescent and under conditions to simulate the microenvironment. These beneficial outcomes have been seen clinically, but were associated with increased immunosuppression and associated infections at the commonly-used drug doses [16]. Based on our results, we would suggest that future clinical studies with idelalisib and chemotherapy should use an initial step-up in drug dosages to determine the optimum regimen for therapeutic efficacy. While a number of mechanisms have been proposed for the synergy, further studies are required to identify the transcriptional changes with IDE in CLL cells in vitro that are required for this activity. Finally, we have demonstrated BCR- and PI3Kδ- independent induction of γH2AX formation via BCR chemo-inhibition in both CLL and solid tumor cells and determine the mechanism for this phenomenon and its biological significance.

## 4. Materials and Methods

### 4.1. Patient Samples, Cell Lines, Culture Conditions 

Patients were selected from the Manitoba CLL Clinic to ensure variation in prior treatments and prognostic markers. B cell isolation from CLL patients was performed as previously described [38,39]. Informed consent was obtained from all participants, and the study was authorized by the human research ethics board at the University of Manitoba (Ethics# HS19803 (H2019:217)). PBMCs were isolated from age-matched volunteers without CLL using the same protocol as for CLL cell isolation except without B cell selection [39]. Freshly isolated cells were used for all experiments and drug exposures were carried out in serum-free hybridoma media (SFM) (Life Technologies, Carlsbad, CA, USA). For the microenvironment simulation experiments, 50 ng/mL each of CD40L and IL4 (R & D Systems, Minneapolis, MN, USA reconstituted in sterile phosphate-buffered saline (PBS), Life Technologies) were added to cells in SFM and allowed to incubate for 1 h prior to drug addition. HeLa, U251MG, and 293T were from American Type Culture Collection (ATCC, Manassas, VA, USA) and were cultured in Dulbecco’s Modified Eagle Medium (DMEM) media (Life Technologies, Carlsbad, CA, USA) and supplemented with 10% fetal bovine serum, 1× Pen/Strep, and 1× Glutamax (Life Technologies). The Burkitt’s lymphoma cell line, BJAB [40], were grown in Roswell Park Memorial Institute (RPMI) 1640 supplemented with 1× Pen/Strep, and 1× Glutamax and 10% fetal bovine serum. Human primary fibroblasts (HPF) were from the Coriell Institute and were cultured in DMEM/F12 media (Life Technologies) and supplemented with 15% fetal bovine serum, 1× Pen/Strep, and 1× Glutamax. Drug exposures for all cell types were carried out at 37 °C and 5% CO_2_ in a humidified atmosphere.

### 4.2. Drugs

BEN, CLB, FLU, and DRB were purchased from Millipore Sigma and IDE and IBR were purchased from Selleckchem (Houston, TX, USA). All agents were reconstituted to 100 mM in dimethyl sulfoxise (DMSO) (MilliporeSigma, Burlington, VT, USA), aliquoted and stored at −80 °C, except DRB which was reconstituted to 50 mM and stored at −20 °C. Aliquots were then diluted in SFM prior to use. DMSO alone treatment was used in all experiments as a negative control and, unless otherwise stated, for normalization.

### 4.3. Flow Cytometry and Drug Synergy

For synergy and dose-response determinations, CLL cells were treated in 96-well plates with 6 increasing concentrations of drug for ~18 or ~72 h. Concentration ranges were selected and optimized to ensure that the exponential phase of the dose-response curve was obtained for the majority of the samples. Ranges were 0–160 µM for BEN, 0–80 µM for IDE and CLB, 0–20 µM for IBR and 0–10 µM for FLU. Synergy experiments with DRB were performed using 3 or 4 increasing doses of drug for ~18 h with the concentration ranges 0–40 µM for BEN and IDE, and 0–20 µM for DRB. The concentration of DMSO was constant between samples. After 18 or 72 h incubation, cell death was determined by flow cytometry by annexin-V-FITC and 7AAD (BD Pharmingen, San Jose, CA, USA) [41]. Cells were stained with for 15 mins with AV/7AAD and analyzed by flow cytometry using a NovoCyte flow cytometer (ACEA Biosciences, San Diego, CA, USA) with a 96-well plate adapter. Cells were considered alive when they were double-negative for AV and 7AAD. When CD19 and CD3 were analyzed in the non-CLL donor PBMCs, anti-CD19-APC or anti-CD3-APC (BD Pharmingen) were added in a triple stain with AV/7AAD. Isotype control antibodies were also run for CD19 (anti-mouse-IgG1κ, BD Pharmingen) and CD3 (anti-mouse-IgG2aκ, BD Pharmingen). 

### 4.4. Animals

C57BL/6 mice (8–10 weeks old) were purchased from and maintained in a pathogen-free facility at the GMC, University of Manitoba, according to the Canadian Council on Animal Care guidelines. Mice were either WT or expressed a mutated inactive form of p110δ (p110δD910A) [42]. Spleens from 2 WT or 4–6 p110δ-deficient mice were crushed, filtered, washed with RPMI (Hyclone, Logan, UT, USA) with 2% Penicillin and Streptomycin (Pen/Strep, Life Technologies, Carlsbad, CA, USA), centrifuged at 4 °C, and resuspended in 2 mL of ammonium-chloride-potassium (ACK) lysis buffer (150 mM NH_4_CL, 10 mM KHCO_3_, and 0.1 mM Na EDTA pH 7.2–7.4) for 2 mins at room temperature. 15 mL Media was then added, samples were centrifuged and resuspended in 1× PBS (Thermo Fisher, Waltham, MA, USA) with 2% FBS (Life Technologies, Carlsbad, CA, USA). B cells were isolated using the EasySep™ Mouse Pan-B Cell Isolation Kit (StemCell Technologies, Vancouver, Canada) as per the manufacturer’s instructions. Isolated splenic B cells were then stimulated for 24 h with CD40L (1µg/mL) and IL4 (5ng/mL) in RPMI with 1% Penicillin/Streptomycin. 2 × 10^6^ cells/mL were then treated with drug, incubated in 100 μL for ~72h at 37 °C and 5% CO_2_ in a humidified atmosphere, and cell death was then measured. Experiments were performed twice with similar results. All mice experiments were approved by University of Manitoba animal care committee (protocol approval number: B2017-0130) under title “Phosphoinositide-dependent signalling pathways controlling B lymphocyte activation” on 1 April 2019 for period from 30 May 2019 to 29 May 2020.

### 4.5. DNA Damage Analysis

DSBs were measured for immunoreactivity to anti-γH2AX antibody after cells were treated for ~18 h with drug. Cellular irradiation (IR; 20 Gy at ~17 h post cell seeding) using a RS-2000 Rad Source (Rad Source Technologies, Inc., Buford, GA, USA) served as a damage-positive control. Thirty min post-irradiation, samples were washed in PBS (MilliporeSigma), resuspended in 70% ethanol (MilliporeSigma), and stored at −20 °C for at least 1 h (up to 4 days). Cells were then washed 3 times with cell staining buffer (Biolegend, San Diego, CA, USA) and 1.75 µL of Alexa 647conjugated anti-H2AXγ antibody or Alexa647-Mouse-IgG1 Isotype control (Biolegend) was added to 50 µL of cell staining buffer at room temperature in the dark for 30 min. Samples were washed and analyzed via a NovoCyte flow cytometer.

Following 24 h treatment with IDE, adherent non-B lineage cells (Hela, U251MG and HPF) were treated followed by immunostaining with 1/1000 diluted Alexa 647-conjugated anti-H2AXγ antibody in 3% bovine serum albumin (BSA)-PBS [43]. Images were captured via epifluorescence microscopy. A minimum of 30 cells per treatment or cell line were scored and the average number of γH2AX-immunostained foci per cell was tabulated and graphed.

### 4.6. Comet Assay

The alkaline comet assay was performed as previously described [43], with the following modifications. Cells were treated for ~18 h. At ~17 h, untreated cells underwent 20Gy irradiation as a positive control for DNA damage. Cells were embedded in agarose on 96-well slides and underwent lysis, alkali unwinding and gel electrophoresis as per manufacturer’s protocols (Trevigen, Gaithersburg, MD, USA). Comets were visualized via SYBR green staining on the Cytation V using Gen5 software (BioTek Instruments, Inc., Winooski, VT, USA). 

### 4.7. IR Recovery, Dose Response, and Synergy 

For synergy and dose-response determination, CLL cells were treated in 96-well plates with 6 increasing concentrations of IDE from 0–80 μM. The concentration of DMSO was constant between samples. Plates were then irradiated with 0, 1, 2, 5, 8, 10 or 20 Gy and cell death and γH2AX were measured after 72 h. In IR recovery experiments, CLL patient cells were stimulated with 50 ng/mL of CD40L and IL4, or with PBS as a control. DMSO or 10 μM of IDE was then added to the cells, the plates were incubated for ~18 h, and the cells were analyzed for γH2AX and comet assay. Simultaneously with the IDE incubation starting at the beginning of drug treatment, at 3 h, at 0.5 h, or immediately prior to γH2AX or comet assay analysis, cells were irradiated with 10 Gy and allowed to recover.

### 4.8. Western Blots

Primary CLL cells were treated with 10 µM IDE and/or 40 µM BEN for ~18 h with or without CD40L/IL4 treatment and pelleted. Protein extracts were prepared and underwent western analysis as per [43]. Blots were immunostained with antibodies listed in Table S1, followed by appropriate horseradish peroxidase–conjugated secondary antibodies (Table S1) and detected using Clarity Western ECL substrate (Bio-Rad, Hercules, CA, USA). Antibodies to actin and vinculin (Table S1), and Ponceau staining (MilliporeSigma) of the transferred membrane were used as protein-loading controls. Blots were visualized using a LAS 500 Imager (GE Healthcare, Chicago, IL, USA). Densitometric analysis was performed via ImageJ. Protein levels were first normalized to a loading control and then phospho-protein levels were normalized to their non-phosphorylated counterparts.

### 4.9. Transcriptional Analysis

Primary CLL cells were treated with either CD40L/IL4 or PBS as a control and incubated for 18 h. 10 μM IDE, 5 μM IBR, 30 μM DRB, or 25 μM BEN were also added either immediately following CD40L/IL4 treatment or after 12 h (for the 6 h drug treatment time point) to cells for a final volume of 100 μL in a 96- well plate. Following drug treatment, the Click-iT^®^ RNA Alexa Fluor^®^ 488 HCS Assay (Life Technologies) was performed as per the manufacturers’ instructions with the following modifications (in consultation with manufacturer). One h prior to the end of drug treatment, 100 μL of 2 mM 5-ethynyl uridine (EU) was added directly to the 100 μL of cells and incubated under normal culture conditions. The cells were then stained for 30 mins at 4 °C with the eBioscience (San Diego, CA, USA) Fixable Viability Dye eFluor™ 780 prior to fixation with 3.7% formaldehyde (MilliporeSigma) and permeabilization with 0.5% Triton^®^ X-100 (MilliporeSigma). After incubation with Click-iT^®^ reaction cocktail and washing with Click-iT^®^ reaction rinse buffer, cells were resuspended in 100 μl of PBS (MilliporeSigma) and analyzed by flow cytometry using a NovoCyte flow cytometer. Cells stained without the viability dye, the Alexa Fluor^®^ azide, or both were run as controls. Click-iT^®^ median fluorescence intensity (MFI) was measured in the viable cells. AV/7AAD was run in parallel as a control for viability. All wash steps were performed in PBS followed by microplate centrifugation at 250 g and removal of the supernatant.

### 4.10. IGHV Mutational Analysis

The immunoglobulin heavy chain variable region (*IGHV)* mutational status of the patients was determined from RNA, as previously described [44]. 

### 4.11. FISH Analysis

Fluorescence in situ hybridization (FISH) analysis was carried out on fresh or stored cells, as previously described [41]. 

### 4.12. Statistical and Synergy Analysis

Graphical representation and statistical analysis were performed using MS Excel and GraphPad Prism. Drug synergy was assessed using Combenefit software [19] and GraphPad Prism [39,45]. Combenefit plots represent the average difference in cell viability or γH2AX production compared to that predicted to the single dose-response curves for each agent (blue—synergy, green—additivity, red—antagonism) [19]. Statistical tables from Combenefit show 95% confidence intervals (* *p* < 5 × 10^−2^_;_ ** *p* < 10^−3^, *** *p* < 10^−4^). Synergy was also assessed according to the method of Chou and Talalay, where the CI is defined as: CI = (d1_x_/D1_x_) + (d2_x_/D2_x_); where d1_x_ and d2_x_ are doses of drug 1 and drug 2, respectively, required to produce a given reduction in cell viability (x) when given in combination, and D1_x_ and D2_x_ are doses of drug 1 and drug 2, respectively, required to produce the same effect in single-agent treatments [46]. CI < 1, =1 and >1 are interpreted as synergy, additivity and antagonism, respectively. The doses chosen for CI analysis were averaged from the closest values available in the drug combination matrix to the clinically relevant dose for each drug (5 μM for IDE, 10 and 20 μM for BEN, 5 μM for CLB, and 2.5 and 5 μM for FLU) [20]. A *p*-value < 0.05 was considered significant.

To determine the correlation between the IC_50_ of the drugs and the average CI values, the Pearson correlation coefficient (r) was calculated and the p-value was determined using a two-tailed T test with a 95% CI. To test the significance of the Click-iT^®^ (Life Technologies) data, a paired two-tailed *t*-test was performed with a 95% confidence interval (* *p* < 5 × 10^−2^_;_ ** *p* < 1 × 10^−2^, *** *p* < 1 × 10^−3^). 

## 5. Conclusions

IDE is a potent inhibitor of the δ isoform of PI3K, a key enzyme in the BCR pathway. IDE has significant clinical efficacy in CLL, but its use is limited by autoimmune colitis, hepatitis and pneumonitis. In the present study, we show significant synergy between IDE and BEN in CLL cells in vitro, both when incubated alone or with CD40/IL4, and this also affects normal B cells but not T cells. These findings emphasize the interaction between the BCR and DNA repair pathways, and the importance for initial dose-escalation studies when combining BCR inhibitors with chemotherapy. 

## Figures and Tables

**Table 1:** Idelalisib (IDE) displays cross resistance with ibrutinib (IBR), not bendamustine (BEN), chlorambucil (CLB) or fludarabine (FLU), resistance to IDE is not predicted by previous treatment of the patient or deletion (del) 17p. The concentration required to inhibit viability by 50% (IC_50_) (blue—sensitivity, red—resistance) and combination index (CI) values (blue—synergy, green—additivity, red—antagonism) from the combination of BEN and IDE at the clinically relevant concentrations (average of 10 and 20 μM for BEN and 5 μM for IDE) of primary chronic lymphocytic leukemia (CLL) cells 72 h post drug treatment.

Sample	IC_50_ (µM)					BEN/IDE CI Value	Patient Characteristics									
	IDE	IBR	BEN	CLB	FLU		Age (years)	Gender	Rai Stage	WBC (× 10^3^/µL)	LDT (Months)	*IgVh* Status	Zap-70 Status	CD38 Status	Previous Treatment	FISH Status (%)
1	1.6	0.6	24.2	14.4	4.9		61	M	II	54	>12	**U**	15%	16%	None	Del 13q14 (81)
2	3.3	1.6	69.1	30.4	>10	1.2	77	F	0	141	>12	M	2.2%	0%	**Yes ^1^**	**Del 17p (43)**, 13q14 (24), ×2 (56)
3	3.7	2.1	9.9	3.4	1.2	0.5	70	M	I	50	>12	M	0%	100%	None	**Tri 12 (90)**
4	4.1	3.8	23.3	7.6	0.7	0.5	60	M	II	89	>12	M	10%	0%	None	Del 13q14 (94)
5	4.9	2.0	38.8	13.5	1.7	0.7	77	M	IV	53	>12	M	37%	45%	None	Del 13q14 (93)
6	5.3	2.4	15.4	10.0	1.2		64	M	II	61	>12	M	3%	45%	None	Normal
7	6.1	3.5	23.8	16.1	3.1	0.4	65	M	II	228	<12	**U**	40%	100%	**Yes ^2^**	**Del 11q (93), Tri 12 (94)**
8	6.6	3.8	15.4	8.8			45	M	I	31	>12	M	1%	0%	None	Normal
9	7.0	3.3	139.4	23.4	4.1	0.5	67	F	III	75	<12	**U**	50%	100%	**Yes ^3^**	**Del 17p (14), 11q (13)**
10	7.6	6.1	6.2	3.8	0.4	0.6	65	M	0	37	>12	M	2.80%	0%	None	Del 13q14 (31)
11	7.8	1.4	12.4	7.6	0.5	0.7	54	M	II	73	>12	M	5%	2%	None	Del 13q14 (25)
12	8.0	1.8	78.9	54.7	>10		60	M	0	75	>12	**U**	N/A	N/A	None	**Del 11q (88)**, 13q14 (92)
13	8.1	3.8	14.0	10.1	7.4	0.6	74	M	I	103	>12	M	N/A	0%	None	Del 13q14 (79)
14	9.0	4.7	14.1	9.1	0.6	0.6	80	F	0	54	>12	M	0%	0%	None	Del 13q14 (15)
15	9.3	2.8	31.9	14.4	4.1	0.5	69	M	II	124	>12	M	3%	3%	None	Del 13q14 (31), ×2 (8)
16	13.2	4.5	23.9	19.3	2.0	0.6	86	M	I	48	>12	M	0%	0%	None	Del 13q14 × 2 (77)
17	13.3	5.5	31.0	26.8	>10	0.7	68	M	II	109	<12	M	6%	1%	None	Del 13q14 × 2 (92)
18	13.5	5.7	51.5	35.5	6.3	0.5	57	F	II	391	<12	**U**	N/A	0%	None	**Del 11q (96)**, 13q14 (98)
19	14.1	3.6	71.5	37.6	>10	0.5	53	M	IV	94	<12	**U**	36%	31%	**Yes ^4^**	Normal
20	14.8	5.0	16.5	8.1	0.6	0.7	64	M	0	52	>12	M	15%	0%	None	Normal
21	15.7	5.2	19.9	13.1	0.7		65	M	II	45	>12	M	21%	42%	None	Normal
22	17.9	4.6	29.8	13.1	1.0		63	M	II	21	>12	M	0%	0%	None	Del 13q14 (90)
23	19.8	3.0	15.4	8.1	0.6	0.4	82	F	0	129	>12	M	3%	7%	None	Normal
24	19.9	5.1	32.4	15.7	1.2	0.6	59	M	I	145	<12	**U**	0%	0%	None	Del 13q14 (92)
25	22.7	6.5	19.8	17.3	>10	0.7	65	M	III	82	>12	**U**	0%	0%	None	**Del 17p (9), 11q (48)**, 13q14 (39), ×2 (20)
26	25.4	6.3	87.6	64.7	>10	0.4	73	F	II	437	<12	**U**	31%	27%	None	Del 13q14 (86)
27	26.5	7.5	134.3	>80	>10	0.8	71	M	II	135	>12	**U**	9%	0%	None	**Del 17p (84)**, 13q14 × 2 (97)
28	27.3	7.0	102.4	65.6	>10	0.6	79	M	III	80	>12	M	7.7%	2%	None	**Del 11q (21)**, 13q14 (93)
29	30.3	5.5	10.3	4.6	1.2	0.7	78	M	I	49	>12	M	4%	0%	None	Del 13q14 (76.19), × 2 (6.19)
30	32.6	3.0	49.2	38.3	>10	0.8	67	M	I	408	<12	M	90%	0%	**Yes ^5^**	Normal
31	45.1	4.4	48.7	24.3	4.1	0.7	91	F	0	30	>12	M	0%	98%	None	**Tri 12 (87)**
32	51.6	9.9	22.7	16.7	1.9	0.6	61	M	I	119	>12	**U**	N/A	N/A	None	Normal
**Med**	13.3	4.1	24.0	14.4	1.2	0.59										

WBC, white blood cell count; LDT, lymphocyte doubling time; M, mutated; U, unmutated; N/A, not available; Tri, trisomy; Del, deletion; x2, double deletion. Treatments of patients in clinic: ^1^ CLB 2004; ^2^ FCR (FLU, cyclophosphamide, rituximab) 2011, obinutuzumab/BEN 2014, IBR 2015; ^3^ BEN 2012; ^4^ FCR 2011; ^5^ FLU 2011, 2013, FCR 2013.

## Supplementary Materials

The following are available online at https://www.mdpi.com/2072-6694/11/10/1519/s1, Table S1: Antibodies used for western blot analysis, Figure S1: Synergy between IDE and BEN was not seen in B cells from mice with non-functional PI3Kδ protein, Figure S2: Induction of γH2AX foci by IDE in non-B cell lineage cell lines, Figure S3: IDE decreases p-AKT levels post stimulation even in the presence of BEN.
