# Supplementary material for: Transcriptional Modulation by Idelalisib Synergizes with Bendamustine in Chronic Lymphocytic Leukemia
Source: Cancers (Basel). 2019 Oct 9;11(10):1519. doi: 10.3390/cancers11101519 (PMC6826782; doi:10.3390/cancers11101519)
Supplement: Supplementary file 1 [file cancers-11-01519-s001.pdf]

Supplementary Materials

Table S1. Antibodies used for western blot analysis.

Antibody	Species	Dilution	Company	Catalogue Number
ATM	rabbit	1:1000	Cell Signalling	2873
phospho-ATM-S1981	rabbit	1:1000	Cell Signalling	13050
TIF1β	rabbit	1:1000	Cell Signalling	4124
phospho-TIF1β	rabbit	1:1000	Cell Signalling	4127
phospho-AKT	rabbit	1:1000	Cell Signalling	9271
AKT	rabbit	1:1000	Cell Signalling	4691
PI3Kδ	rabbit	1:1000	Cell Signalling	34050
BTK	rabbit	1:1000	Cell Signalling	8547
anti-rabbit	goat	1:2000	Bio-Rad	170-6515
anti-mouse	goat	1:2000	Bio-Rad	170-6516
actin	rabbit	1:2000	Sigma	A2066
vinculin	mouse	1:10,000	Abcam	ab18058

ATM: ataxia telangiectasia mutated, TIF1β: transcription intermediary factor 1-β, AKT: protein kinase B, PI3Kδ: phosphatidyl-inositol 3 kinase δ, BTK: Bruton's tyrosine kinase.

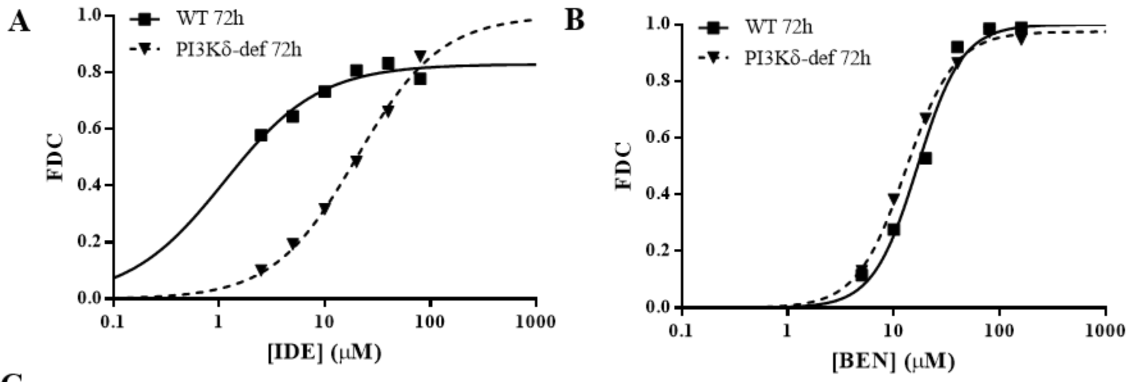

C

	IC50 (μM) at 72h					BEN/IDE CI value	
	IDE	IBR	BEN	CLB	FLU	18h	72h
WT	1.7	2.1	16.7	4.3	5.4	0.73	0.60
PI3Kδ-def	20.1	3.1	13.3	4.3	7	0.93	0.95

Figure S1. Synergy between IDE and BEN was not seen in B cells from mice with non-functional PI3Kδ protein. Splenic B cells were isolated from wild-type (WT) DO11-10 mice and mice lacking functional p110δ PI3K. Cells were stimulated for 24 h with CD40L/IL4, then drugs were added for 18 or 72 h and cell death was measured. (**A**,**B**) Single agent dose-response comparing response of B cells from the PI3Kδ-deficient (PI3Kδ-def) and WT mice treated with singe agent IDE (**A**) or BEN (**B**). (**C**) Table showing differences in response of PI3Kδ-def and WT mouse B cells to single agent drugs or BEN/IDE CI value at the clinically relevant concentrations for humans 18 and 72 h. Results are representative of 2 experiments.

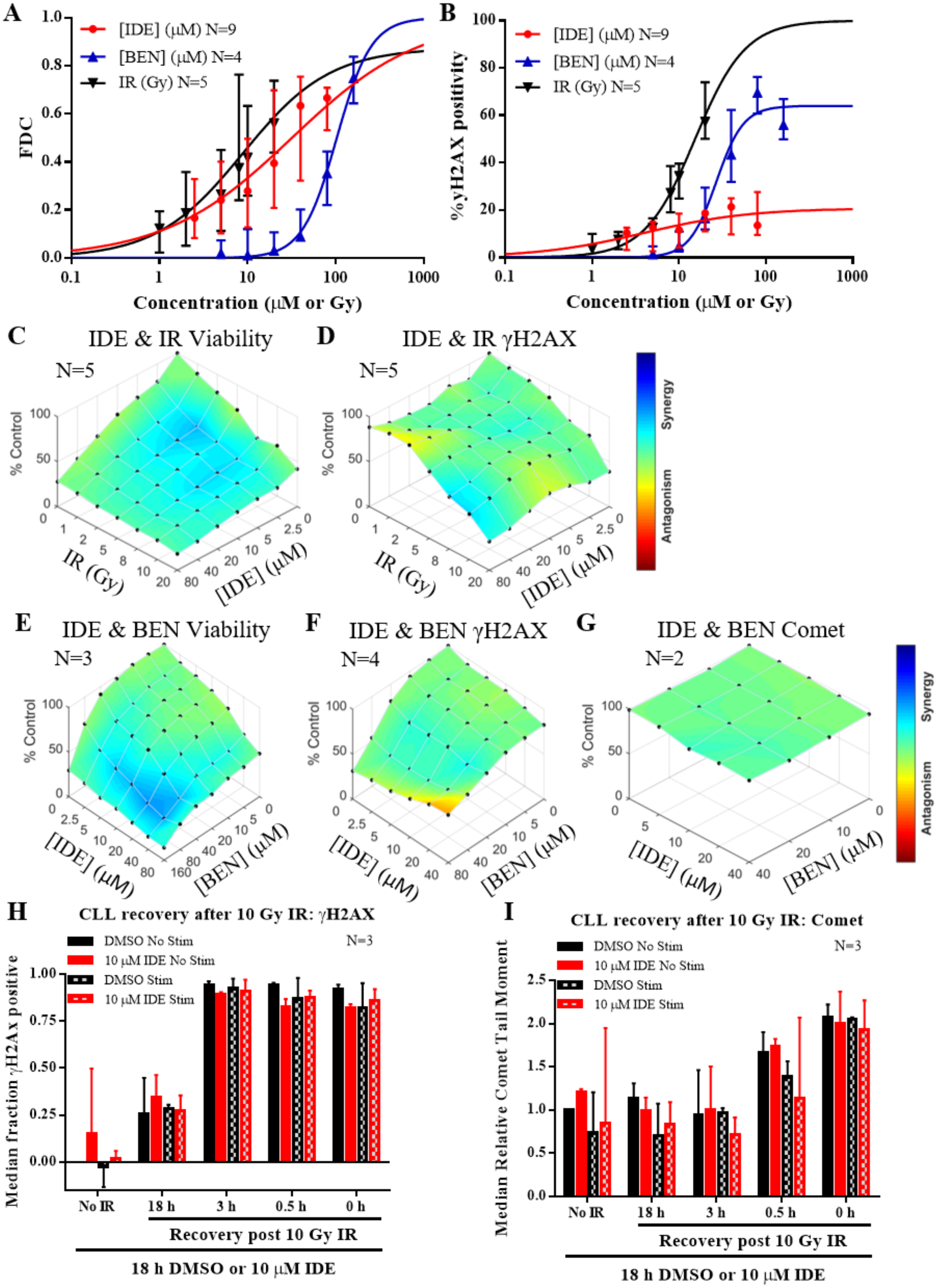

Figure S2. BEN and IR produce more γH2AX than IDE, however neither BEN nor IDE produce DNA breaks. IDE is synergistic with IR and BEN through apoptosis, not γH2AX production, and recovery post IR is not influenced by IDE. (**A**–**G**) Apoptosis or DNA damage responses were measured in primary CLL samples by flow cytometry for AV/7AAD or γH2AX and the comet assay, respectively.

(**A**,**B**) Median single agent 18 h dose-response curves with interquartile range of viability (**A**) or γH2Ax positivity (**B**). C-G. Combenefit synergy plots representing the difference in viability (**C**,**E**), γH2Ax positivity (**D**,**F**), or comet tail moment (relative to 20 Gy IR, **G**) from what was expected from the single dose-response curves when IDE was combined with IR (**C**,**D**) or BEN (**E**–**G**). Blue - synergy, green – additivity, and red – antagonism. Some data points were removed from γH2AX plots at the high concentrations if the cell death was too high to interpret the γH2AX staining. (**H**) Graphs of the recovery of primary CLL cells treated with DMSO or 10 μM IDE for 18 h prior to being analyzed for γH2AX positivity. CLL cells were incubated with 10 μM IDE for 18 h, either alone or combined with CD40L/IL4, as previously described [23]. During the 18 h treatment period, cells were treated with 10 Gy IR at different times and allowed to recover for 18, 3, 0.5 hrs, or not at all. Without IR, IDE induced more γH2AX than DMSO treated cells, and there was more γH2AX when cells were incubated alone than with CD40L/IL4. While γH2AX levels were minimally-changed 3 h post-IR, 75% of the γH2AX had disappeared by 18 h and the rate of loss was independent of IDE. (**I**) Graphs of the recovery of primary CLL cells treated with DMSO or 10 μM IDE for 18 h prior to being analyzed via comet tail moment. Cells were treated with 10 Gy IR at the beginning of drug treatment, 3 h, 0.5 h and immediately prior to analysis. Graphs show median and interquartile range.

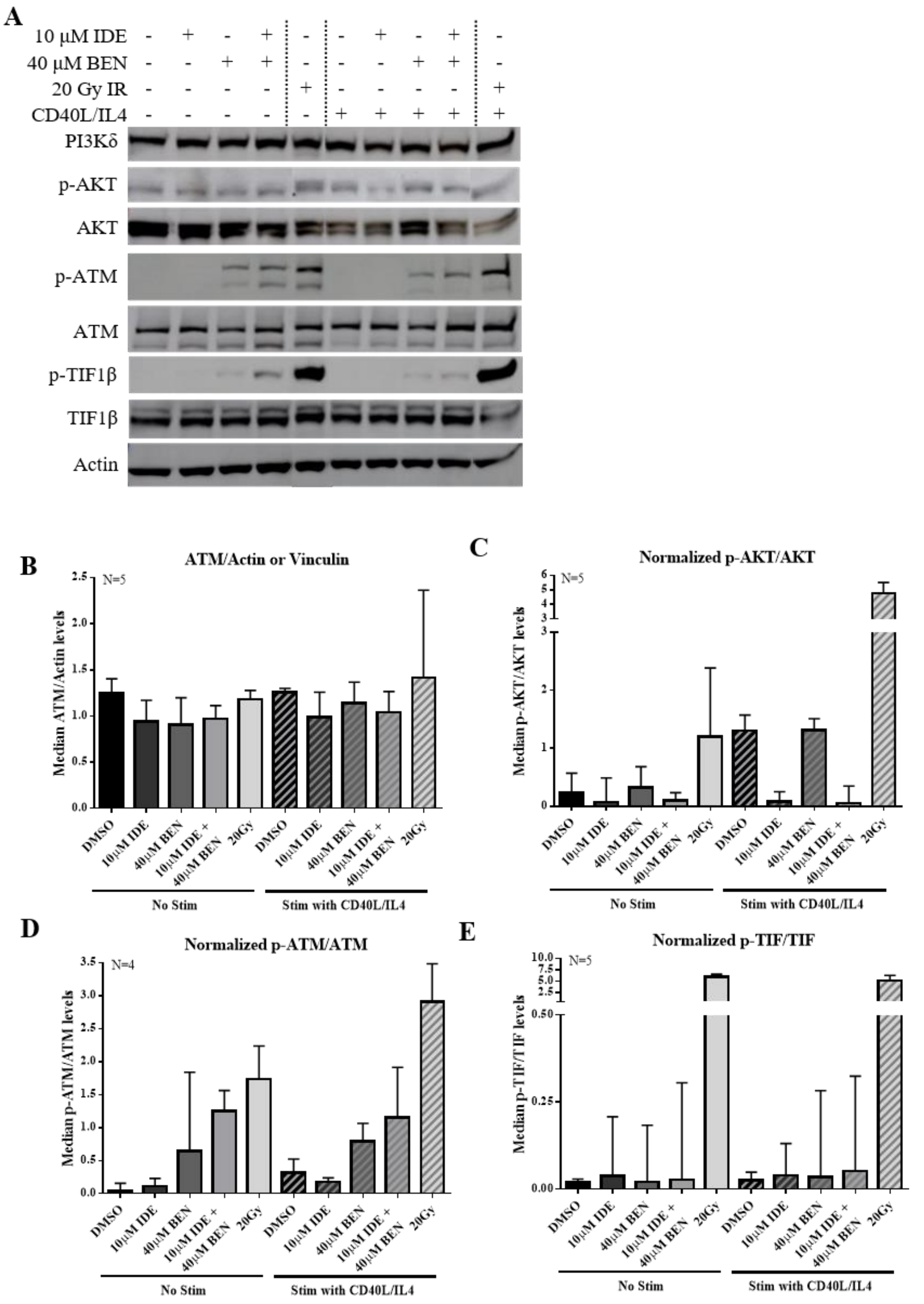

Figure S3. IDE decreases p-AKT levels post stimulation even in the presence of BEN. DNA damage response proteins are increased by BEN, even in the presence of IDE. Cell pellets were made from 7 unique primary CLL patients 18 h post drug treatment with and without stimulation (Stim) with CD40L/IL4. (**A**) Representative western blot probed for proteins important in the action of IDE (PI3Kδ and AKT) and DNA damage proteins (ATM and TIF1β). Picture was made from the same blot and dashed lines represent where irrelevant samples were removed. (**B–D**) Median densitometry with interquartile range of ATM (**B**), p-AKT (**C**), p-ATM (**D**), or p-TIF (**E**). Protein levels were first normalized to a loading control and then phospho-protein levels were normalized to their non-phosphorylated counterparts. 20 Gy IR with 30 min recovery was used as a positive control.
